# Higher increase degree of FGF21 post long-term interdisciplinary weight loss therapy preserves the free fat mass and rest metabolic rate in adolescents with obesity

**DOI:** 10.20945/2359-3997000000224

**Published:** 2020-03-30

**Authors:** Ana Claudia Pelissari Kravchychyn, Raquel Munhoz da Silveira Campos, Yasmin Alaby Martins Ferreira, Sofia Emanuelle de Castro Ferreira Vicente, Flávia Campos Corgosinho, Lila Missae Oyama, David Thivel, Lian Tock, Ana Raimunda Dâmaso

**Affiliations:** 1 Programa de Pós-graduação em Nutrição Universidade Federal de São Paulo São Paulo SP Brasil Programa de Pós-graduação em Nutrição, Universidade Federal de São Paulo, São Paulo, SP, Brasil; 2 Universidade Federal de São Paulo Campus Baixada Santista Santos SP Brasil Universidade Federal de São Paulo, Campus Baixada Santista, Santos, SP, Brasil;; Departamento de Biociências Programa de Pós-graduação Interdisciplinar em Ciências da Saúde Universidade Federal de São Paulo Santos SP Brasil Departamento de Biociências, Programa de Pós-graduação Interdisciplinar em Ciências da Saúde Universidade Federal de São Paulo, Campus Baixada Santista, Santos, SP, Brasil; 3 Universidade Federal de Goiás Goiás SP Brasil Universidade Federal de Goiás, Goiás, SP, Brasil; 4 Université Clermont Auvergne Clermont Ferrand France Université Clermont Auvergne, Clermont Ferrand, France

**Keywords:** Body composition, obesity, adolescents, FGF21, weight loss, thermogenesis

## Abstract

**Objective:**

Fibroblast growth factor 21 (FGF21) is among the activators that can stimulate thermogenesis in the white adipose tissue and brown adipose tissue. People with obesity have elevated blood levels of FGF21, but also develop resistance to its action, impairing its beneficial role. Inversely, clinical treatments to weight loss has been pointed out as an important therapy for increasing and recovering sensitivity to FGF21. The aim was to analyse the effect of long-term weight loss interdisciplinary intervention on FGF21 and body composition.

**Subjects and methods:**

Eighty-six post-pubertal obese adolescents (14-19 years-old), were submitted to 20 weeks of weight loss therapy (clinical, nutritional, psychological and physical exercise support). Anthropometric measures, body composition and rest metabolic rate (RMR) by bioelectrical impedance, and serum FGF21 sample by ELISA were evaluated. The adolescents were grouped according to FGF21 individual delta variations after therapy: Higher Increase (HI); lower increase (LI); lower decrease (LD); higher decrease (HD).

**Results:**

All groups present weight loss. Only in FGF21 ≥ 76,5 pg/mL variation the free-fat-mass and rest metabolic rate were preserved and to others group these variables were significantly reduced.

**Conclusion:**

High increase in FGF21 can contribute to preservation of FFM and RMR after weight loss therapy, could have important implications for energy balance regulation. Future studies are necessary to continue determining the role of magnitude effects of FGF21 levels in obesity to improve clinical practice, especially in paediatrics population.

## INTRODUCTION

The fibroblast growth factor 21 (FGF21) is among the activators that can stimulate thermogenesis in the white adipose tissue and brown adipose tissue (BAT), giving rise to beige cells through the Uncoupling Protein-1 activation, and consequently for mitochondrial biogenesis. Human stress conditions such as exercise, obesity, insulin resistance and diabetes can influence FGF21 serum levels and action ( 1 - 3 ).

People with obesity have elevated blood levels of FGF21 but also develop resistance to its action, impairing its beneficial action ( 2 ). In contrast, physical exercise has been pointed out as an important therapy for increasing and recovering sensitivity to FGF21 ( 2 , 4 ).

The direct relationship between BAT and muscle mass in this population of adolescents it has been elucidated, while high FGF21 levels observed in adolescents with obesity were positively correlated with liver and visceral fat levels ( 5 ). Further studies seem then necessary to assess the effects of weight loss (WL) interventions on FGF21 as a thermogenesis marker in adolescents with obesity. The aim of the present investigation was to analyse the effect of long-term WL interdisciplinary intervention on FGF21 and the relationship with body composition (BC) and individual variability of FGF21.

## SUBJECTS AND METHODS

This study involved 86 obese adolescents of both genders, 14 to 19 years, post-pubertal Stage ≥V ( 6 ) and body mass index (BMI) >95^th^ percentile according Word Health Organization curves. The non-inclusion criteria were: pregnancy, previous drug utilization, chronic alcohol consumption, viral hepatic diseases, other causes of liver steatosis, inability to perform physical activities. The study was conducted with Declaration of Helsinki and was approved by ethics committee (#0052/2016) and clinical trials (RBR-6txv3v).

### Research design

The classical interdisciplinary therapy consisted of clinical assessment, exercise training, nutritional and psychological support for 20 weeks. The protocol also used a web-based approach regarding different health education themes. The adolescents visited the endocrinologist to address their health conditions and determine sexual maturation at baseline and after therapy.

Six interdisciplinary clinical interventions were performed. They had low-calorie dietary prescriptions per age and a gender. The distribution of macronutrients was fat (25-35%), carbohydrate (45-65%) and protein (10-30%) ( 7 ). Every week different health themes were posted in online weight loss program with dietetics lessons educating (Example: low-calorie foods, diet and light foods, weight loss diets, good food choices on holidays, weekends and celebrations, food labels and other related topics).

Physical exercise was chosen by the self-guided method in which the adolescent opted for exercises related to personal pleasure ( 8 ). The choice was guided in all clinical approach by a professional in this area for frequency (tree times/weekly) and duration (minimum of one hour/session) and were considered variables of body composition and basal metabolic rate to the choice of the modality to be practiced guaranteed benefits to the weight loss program ( 9 ). In the online program health themes, the volunteers had access to videos about correct mode of physical exercise practice including frequency, intensity and volume to help them in their choices.

The adolescents participated in group-based psychological sessions to help them deal with themes of depression, disturbances of body image and anxiety ( 10 ).

### Anthropometric measurements, body composition and serum analysis

At baseline and after treatment, body mass (Filizola^®^) and height (Sanny^®^) were measured and BMI was calculated. BC was assessed using bioelectrical impedance (TBW^®^) providing Body Fat (BF), Free Fat Mass (FFM) and estimating Rest Metabolic Rate (RMR). Serum levels of FGF21 were measured with enzyme-linked immunosorbent assay.

### Statistical analysis

Statistical analysis was performed using STATISTICA 13.0 software (StartSoft, Tulsa OK. USA). Statistical significance set at p < 0.05. Data normality was verified with the Kolmogorov-Smirnov test. Parametric data were expressed as mean ± SD while nonparametric variables were normalized by Z score. Comparisons between measures at baseline and after by groups were made using ANOVA repeat measures with Turkey post-hoc.

## RESULTS

**Figure 1:**
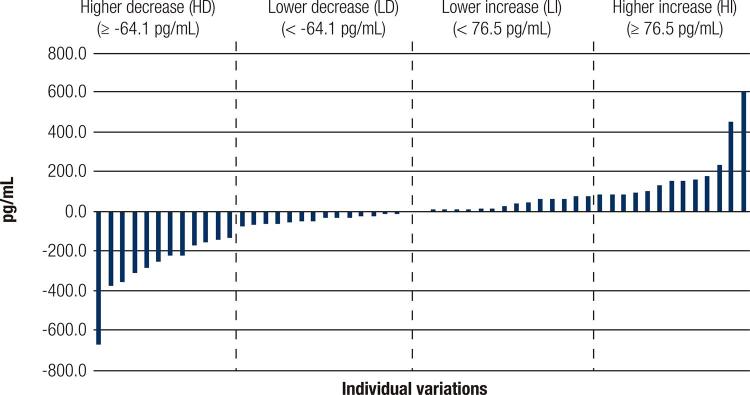
Individual variations of FGF21 serum levels after interdisciplinary therapy to weight loss. FGF21 were expressed in pg/mL. Lower increase (LI) < 76.5 pg/mL FGF21 (n = 13); higher increase (HI) ≥ 76.5 pg/mL FGF21 (n = 14); higher decrease (HD) < -64.1 pg/mL FGF21 (n = 14); and lower decrease (LD) ≥ -4.1 pg/mL FGF21 (n = 14).

A total 56 patients completed the intervention and four groups were determinate according to median of variations (deltas) in FGF21 levels (pg/mL): Increase: < 76.5 and ≥ 76.5; Decrease: < -64.1 and ≥ -64.1 ( Figure 1 ).

All groups present weight loss. The BMI was significantly reduced only in the group with Increase FGF21 ≥76.5 and BF reduced just in the group that presented Decreased FGF21 ≥ -64.1. Considering FFM and RMR, exclusive in the group that Increased FGF21 ≥ 76,5 these variables did not suffer significant reduction ( Table 1 ).

**Table 1:** Descriptive data of body composition on different groups of FGF21 increased and decreased in obese adolescents submitted to multidisciplinary therapy

	Increase FGF21					

	< 76.5 pg/mL (n = 13)			≥ 76.5 pg/mL (n = 14)		
	
	Baseline	After	P	Baseline	After	P
Body weight (kg)	112.4 ± 20.7	105.7 ± 18.2^†^	0.00	111.6 ± 12.8	107.0 ± 12.6^†^	0.04
BMI (kg/m^2^)	37.3 ± 5.3	34.9 ± 5.1	0.53	39.1 ± 4.4	37.9 ± 4.6^†^	0.02
Body Fat (kg)	39.5 ± 10.2	35.0 ± 8.8	0.06	44.3 ± 7.1	41.2 ± 6.9	0.40
FFM (kg)	72.6 ± 13.4	70.7 ± 12.9^†^	0.01	70.8 ± 9.3	69.8 ± 9.3	0.85
RMR (kcal)	2207.7 ± 407.1	2148.5 ± 391.1^†^	0.00	2152.8 ± 284.5	2124.9 ± 281.7	0.88
FGF21	187.5 ± 143.3*	226.8 ± 137.9^†^	0.01	97.9 ± 135.5*	271.4 ± 256.6^†^	0.00

	**Decrease FGF21 **					

	**< -64.1 pg/mL (n = 14)**			**≥ -64.1 pg/mL (n = 14)**		
	
	**Baseline**	**After**	**P**	**Baseline**	**After**	**P**

Body weight (kg)	107.2 ± 13.9	101.9 ± 13.5^†^	0.00	112.9 ± 15.8	104.9 ± 14.9^†^	0.00
BMI (kg/m^2^)	37.5 ± 5.1	35.4 ± 5.3	0.91	39.5 ± 4.1	36.7 ± 3.8	0.25
Body Fat (kg)	40.2 ± 7.0	36.8 ± 6.6	0.28	45.2 ± 9.5	39.3 ± 7.7^†^	0.00
FFM (kg)	66.9 ± 10.8	65.1 ± 10.1^†^	0.00	67.7 ± 10.1	65.6 ± 10.2^†^	0.00
RMR (kcal)	2034.8 ± 331.9	1979.6 ± 307.5^†^	0.01	2059.3 ± 308.5	1989.7 ± 300.5^†^	0.00
FGF21	183.9 ± 165.7*	152.7 ± 168.1	0.89	426.0 ± 261.0	182.0 ± 143.0^†^	0.00

*p < 0.05 compared to decrease FGF21 ≥ -64.1; ^†^p < 0.05 compared to baseline in the same group. FGF21 were expressed in pg/mL. BMI: body mass index; FFM: free fat mass; RMR: rest metabolic rate.

## DISCUSSION

The aim of the present investigation was to assess the individual variability of FGF21 concentration and its impact on BC in adolescents with obesity undergoing long-term interdisciplinary WL therapy. In this context, when analysing variation degrees, it was observed that in the group with increase FGF21 ≥ 76.5 seems to preserve the FFM and RMR compared to another groups.

The evidences questioning the anti-obesity effects of FGF21 in adolescent with obesity remains scarce, while an association between increased circulating FGF21 and metabolic disorders has been reported in adults. Cross-sectional studies conducted in adolescents with obesity showed controversial results regarding the associations between FGF21, BC, metabolic parameters and inflammatory profile ( 3 , 11 - 13 ).

Based on the present results, we can observe a broad variation of FGF21 levels at baseline and individual responses post treatment. Although weight and BF responses have not been different between groups, the FFM and RMR did not reduce only in high increase degree of FGF21.

The FFM and RMR could have important implications for energy expenditure, energy balance regulation and WL maintenance in youth with obesity. In this way, the FFM and RMR preservation post WL treatment with high increase of FGF21 contributes to initial investigate relationship between these variables. In a large cohort, Li and cols. ( 12 ) observed that lower FGF21 levels were negatively correlated with BMI and waist circumference and associated with obesity compared with normal-weight patients.

In addition, it has recently been demonstrated by our group positive correlation between FGF21 and lean body mass and HDL cholesterol in adolescents without insulin resistance, linking the potential effects of FGF21 with glucose metabolism ( 3 ). Markan ( 14 ) suggests that the role in increased circulating FGF21 during obesity remains uncertain and the translating rodent studies to man needs to be done with caution. Human FGF21 is proteolytically cleaved in vivo like this; the bioactivity of increased circulating FGF21 in humans remains unknown.

This study presents some limitations such as the small sample size post-treatment and lack of a lean control group. However, the present data seeks to understand the undersupply of studies that describe FGF21 in humans, especially in early ages.

In conclusion, the present study suggests that increase in FGF21 serum levels can contribute to preservation of FFM and RMR after interdisciplinary therapy to WL in adolescents with obesity. Future studies are necessary to continue determining the role of FGF21 levels in obesity to improve clinical practice, especially in paediatrics population.
